# How well can rock avalanche runout be forecasted? GPU-Accelerated validation of a method for forecasting rock avalanche motion using a numerical model

**DOI:** 10.1007/s10346-026-02831-9

**Published:** 2026-08-26

**Authors:** Guotong Deng, Jordan Aaron

**Affiliations:** Geological Institute, Department of Earth and Planetary Sciences, ETH Zürich, 8092 Zurich, Switzerland

**Keywords:** Rock avalanche, ORIN-3D, Probabilistic forecast, Probabilistic calibration, Tversky loss function

## Abstract

Rock avalanches are extremely rapid, flowlike landslides that can threaten people and infrastructure, making accurate forecasts of their motion using numerical models critical for risk assessment. These forecasts are a primary input into land use and evacuation decisions; however, a comprehensive analysis of the effectiveness of various forecasting approaches applied to a wide variety of rock avalanche types has never been done. This is primarily due to a lack of field data, as well as the prohibitive computational cost. In this work, we address both limitations by applying a Bayesian posterior analysis and leave-one-out cross validation strategy to a dataset of 22 case histories. This enables us to calibrate the GPU (Graphics Processing Unit)-accelerated runout model ORIN-3D (parallel simulatOR of landslIde motioN over 3D terrain) for three rheological parameterizations: (1) Frictional, (2) Voellmy, and (3) a combined Frictional-Voellmy rheology. The analysis involves around 400,000 simulations, which is a computational effort that would be impractical with CPU (Central Processing Unit)-based models. Probabilistic forecasts for each case history are conducted using calibration results across eight forecast sets and evaluated with the Tversky loss function (TLF). The results show that the best performing model parameterization uses a volume-dependent friction angle in the source zone (Frictional rheology) and Voellmy rheology in the path zone. This work thus provides systematically validated, probabilistic forecasts of a database of 22 rock avalanche case histories, enabling improved hazard and risk assessments of this damaging phenomenon.

**Supplementary Information:**

The online version contains supplementary material available at https://doi.org/10.1007/s10346-026-02831-9.

## Introduction

**Fig. 1:**
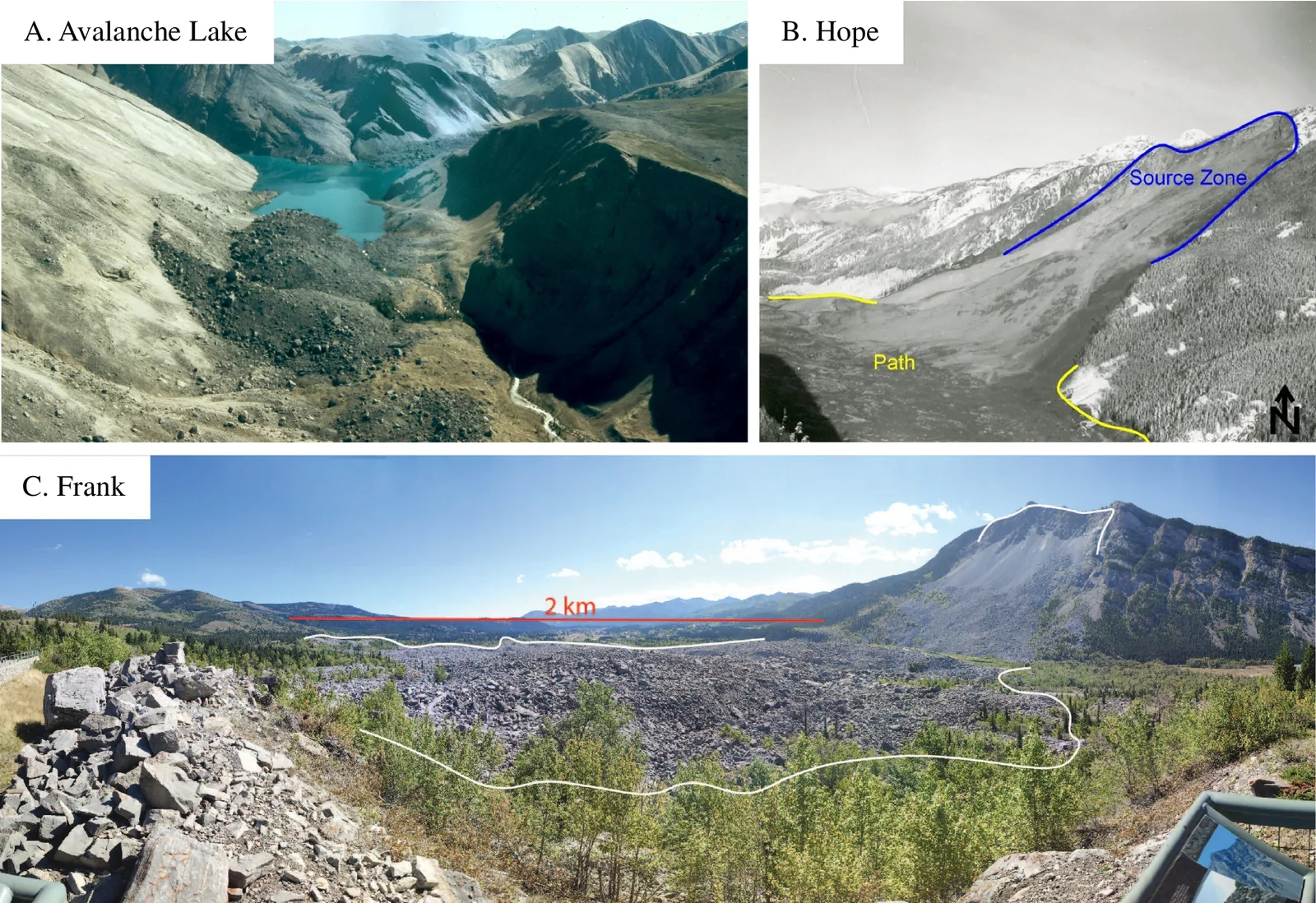
Overview of rock avalanches: **A** Avalanche Lake, **B** Hope, **C** Frank. **A** Modified after Aaron and Hungr (2016a, b), **B** courtesy British Columbia Ministry of Transportation and Infrastructure. **C** Jordan Aaron

Rock avalanches are extremely rapid flows of fragmented rock that can cause casualties and damage infrastructure over wide spatial areas (e.g.,Evans et al. 1994; Hungr 2006; Hungr et al. 2014; Dufresne et al. 2016; Aaron and Hungr 2016a; Aaron and McDougall 2019). Prior to the initiation of a rock avalanche, precursory slope displacements are often observed, which provides an opportunity for practitioners and decision-makers to make probabilistic assessments of the associated hazard and risk (e.g.,Fell et al. 2005; Hungr 2016; McDougall 2017; Aaron et al. 2022). Despite decades of work on forecasting rock avalanche motion, there are still open questions regarding how to parameterize numerical models for forecasts, as well as how reliable the resulting forecasts are. Addressing these uncertainties has been critically limited by the computational efficiency of traditional CPU-based models, as well as a lack of 3D input data for a large sample of rock avalanche case histories. The present work leverages a database of 22 rock avalanche case histories (overviews of parts of the case histories are shown in Fig. 1) and high-performance computing on GPUs to calibrate and cross-validate forecasts of rock avalanche motion. This enables the first systematic quantification of the uncertainties associated with forecasting rock avalanche motion and identifies the most appropriate model configuration and parameters to use for forecasting.

Landslide forecast methods can be divided into data-driven and physics-based methods (Gong et al. 2024). In data-driven methods, mathematical or machine learning models are built to characterize and forecast landslide motion based on historical data using methods such as regression analysis (Wang et al. 2023), artificial neural networks (Zhang et al. 2022), and support vector machines (Gong et al. 2022). For rock avalanche, a commonly used data-driven approach is to use the H/L ratio of a potential landslide to quantify runout, which is the ratio of fall height to runout length. In these approaches, variables such as volume, topographic confinement, and path material type are treated as regression parameters, and databases containing H/L measurements are then analyzed to develop functions for forecasting (Hsu 1975; Whittall et al. 2017; Mitchell et al. 2020). These data-driven approaches work well for preliminary screening; however when more detailed analyses are required, physics-based numerical models are typically used. In particular, Aaron et al. (2022) compared statistical and numerical approaches and found that, if the physics-based numerical model is appropriately parameterized, it can provide forecasts with narrower confidence bands.

Physics-based numerical models differ in dimensionality, numerical solution methods, and parameterization. The most complex models are either continuum or discontinuum 3D models, which can accurately capture internal shear of the landslide mass (e.g.,Johnson et al. 2016; Song et al. 2021; Roshan and Yerro 2025). These models are useful for exploring many important mechanisms related to micromechanics; however, long model runtimes limit their use for hazard and risk analysis. Depth-averaged, continuum based numerical models provide a good tradeoff between simulation time and physical complexity, which makes their use more practical in scenarios where lots of model runs are required, such as model calibration and probabilistic forecasts (e.g.,Hungr 1995; Bouchut et al. 2003; McDougall and Hungr 2004; Christen et al. 2010; Aaron and Hungr 2016b; Mergili et al. 2018).

In the present work, a model that is based on the “equivalent fluid” framework is used. This model treats the rock avalanche mass as a fluid whose behavior is governed by simple internal and basal rheologies (Hungr and McDougall 2009). These kinds of models have been applied and validated for a large number of case histories (e.g.,Quan Luna 2012; Aaron and McDougall 2019) and have seen widespread use in practice. However, since the parameters can only be derived through back-analysis of past case histories, there is still substantial uncertainty regarding how best to parameterize these models for making forecasts, and the errors associated with these forecasts have never been explicitly quantified.

Forecasts of rock avalanche motion are most usefully performed using probabilistic methods due to the uncertainty associated with selecting input parameters. Some previous studies have developed and applied such methods, such as a Bayesian posterior analysis (Aaron et al. 2019), Markov chain Monte Carlo simulation (Zeng et al. 2021), polynomial response surface method coupled with the first-order reliability method (Sun et al. 2021b), and multi-objective optimization inversion analysis (Zeng et al. 2023). These methods require tens of thousands of repeated numerical model runs, which have previously made them impractical and have limited our ability to quantify associated uncertainties. To address this limitation, methods such as Kriging (Zeng et al. 2021; Sun et al. 2021a), active learning (Zhao and Kowalski 2022), and the probability density evolution method (Sun et al. 2023) are used to drastically reduce the number of numerical model simulations required while maintaining accuracy. Nevertheless, these solutions may introduce additional uncertainties, and they have not yet been applied to assess the performance of different rheological parameterizations in a forecasting framework. Furthermore, they have never been applied to systematically analyze a database of rock avalanche case histories, so the best performing probabilistic forecasting method, as well as associated uncertainties, has yet to be understood.

These uncertainties can be subdivided into two types: (1) those associated with calibration and model assumptions (calibration uncertainty) and (2) those caused by unknowns in the mechanisms that govern rock avalanche motion (mechanistic uncertainty) (Aaron et al. 2022). Calibration uncertainties can be quantified using probabilistic calibration procedures (e.g., Aaron et al. 2019) and mechanistic uncertainties through different methods of clustering rock avalanche case histories (Aaron and McDougall 2019; Aaron et al. 2022). However, the influence of these uncertainty types on forecasts of rock avalanche motion still remains unclear. This is because typical runtimes of depth-averaged models are on the order of tens of minutes, meaning that years to decades of simulation times are needed in order to fully explore uncertainties associated with calibration and forecast. Recently, Aaron (2023) proposed the model ORIN-3D, which is a depth-averaged equivalent fluid model optimized to run on the GPU. This model has simulation runtimes on the order of a few seconds, which has enabled the large-scale uncertainty analysis performed herein.

The present paper is arranged as follows. In the “Data” section, we describe the dataset used. Next, in the “Methods” section, we describe the calibration and forecast methodology, as well as the novel evaluation approach. We evaluate forecast performance in the “Results” section and highlight the best performing methodologies as well as remaining uncertainties in the “Discussion” section.

## Data

The database used in this work contains a diverse sampling of 22 rock avalanche case histories (Table 1). For example, it contains the extraordinarily mobile Avalanche Lake rock avalanche, which created a runup of over 500 m (Fig. 1A, Aaron and Hungr 2016a). It also includes the Crammont rock avalanche (Deline et al. 2011), which had a small volume (~ 300,000 m^3^), but experienced high mobility, likely due to mixing with snow. Furthermore, we analyze events such as the Daubensee and Sentinel rock avalanches (Castleton et al. 2016; Grämiger et al. 2016), which had limited mobility despite their large volumes. Taken together, the full dataset represents a broad sampling of the diversity of rock avalanche emplacement characteristics (Table 1), and they therefore serve as a useful validation dataset for quantifying forecasting uncertainties.

**Table 1:** Mobility and path materials of case histories

	**Name**	**Volume (m**^**3**^**)**	**H/L**	**Path materials**
1	Avalanche Lake	192,610,253	0.160	Saturated
2	Chisca	1,000,000	0.241	Saturated
3	Crammont	360,000	0.512	Glacier
4	Daubensee	5,140,000	0.377	Bedrock
5	Frank	40,000,000	0.250	Saturated
6	Goldau	37,473,299	0.210	Saturated
7	Hope	47,300,000	0.398	Saturated
8	Madison Canyon	20,000,000	0.270	Unsaturated
9	McAuley	7,323,341	0.250	Saturated
10	Mt Meager	48,500,000	0.171	Saturated
11	Mt Steele	31,496,054	0.321	Glacier
12	Mystery Creek	17,538,380	0.313	Saturated
13	Platten	11,000,000	0.382	Saturated
14	Rautispitz	116,000,000	0.320	Saturated
15	Rinderhorn	37,000,000	0.241	Saturated
16	Sentinel	286,222,592	0.361	Unsaturated
17	Six des Eaux Froides	5,000,000	0.281	Saturated
18	Thurweisser	2,184,689	0.479	Bedrock
19	Turnoff Creek	4,000,000	0.281	Saturated
20	Val Pola	49,607,100	0.339	Saturated
21	West Salt Creek	11,000,000	0.140	Saturated
22	Zymoetz	900,000	0.310	Saturated

We tested the generalizability of the dataset, as Aaron et al. (2022) showed that these cases appear to be biased towards high mobility on average. We therefore tested whether our database contains cases with anomalous mobility, using published compilations of rock avalanche mobility statistics, which were derived from a larger database encompassing multiple landslide types (Corominas 1996) and a larger database only with rock avalanches (Mitchell et al 2020). To detect outliers, each case in our database was compared with the empirical volume–H/L relationships from all cases in the two reference databases. Standardized residuals were calculated as the difference between the observed and predicted log(H/L) values divided by the reported standard error of the regression. Cases with an absolute standardized residual greater than 2 were classified as mobility outliers relative to the general empirical trend. Using this criterion, only one case, the Sentinel rock avalanche, was identified as a mobility outlier. It plots above the regression line, indicating lower mobility than expected for its volume. This characteristic should be considered when interpreting the subsequent analyses.

## Methods

**Fig. 2:**
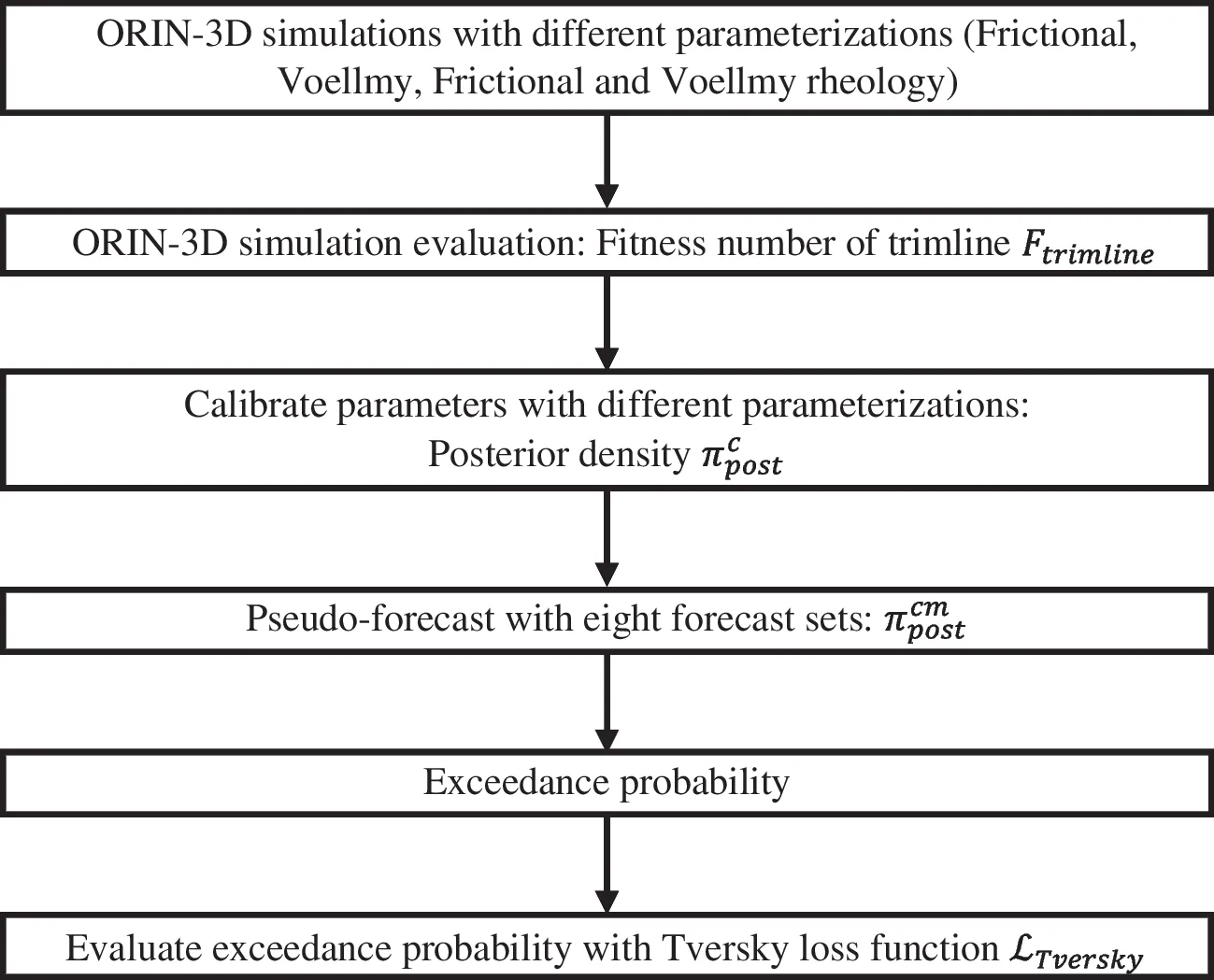
Flowchart showing the methodology used in the present work, which evaluates forecast performance that accounts for both calibration and mechanistic uncertainty through a leave-one-out cross validation approach

In the present work, uncertainties associated with forecasting rock avalanche runout are quantified by adopting a methodology that propagates uncertainties from calibration, through parameterization, to forecasting (Fig. 2). First, the numerical model ORIN-3D used in this work is introduced in the first subsection in this section. Probabilistic calibration of all case histories in the dataset using three different model parameterizations is first performed, and the methodology is detailed in the subsection “Calibration framework.” Then, a leave-one-out cross validation strategy is used to generate pseudo forecasts of each case history using a variety of model parameterizations and different weighting methods for combining the calibration results, which would all result in different forecasting performance. The corresponding methodology is presented in the subsections “Forecast framework,” “Case weightings,” and “Forecast sets.” Finally, the Tversky loss function is introduced in the final subsection and is used to quantify the quality of the pseudo forecasts, in order to understand the optimal forecast methodology and associated uncertainties. The methods used for each of these steps are described below.

### Overview of the numerical model ORIN-3D

**Fig. 3:**
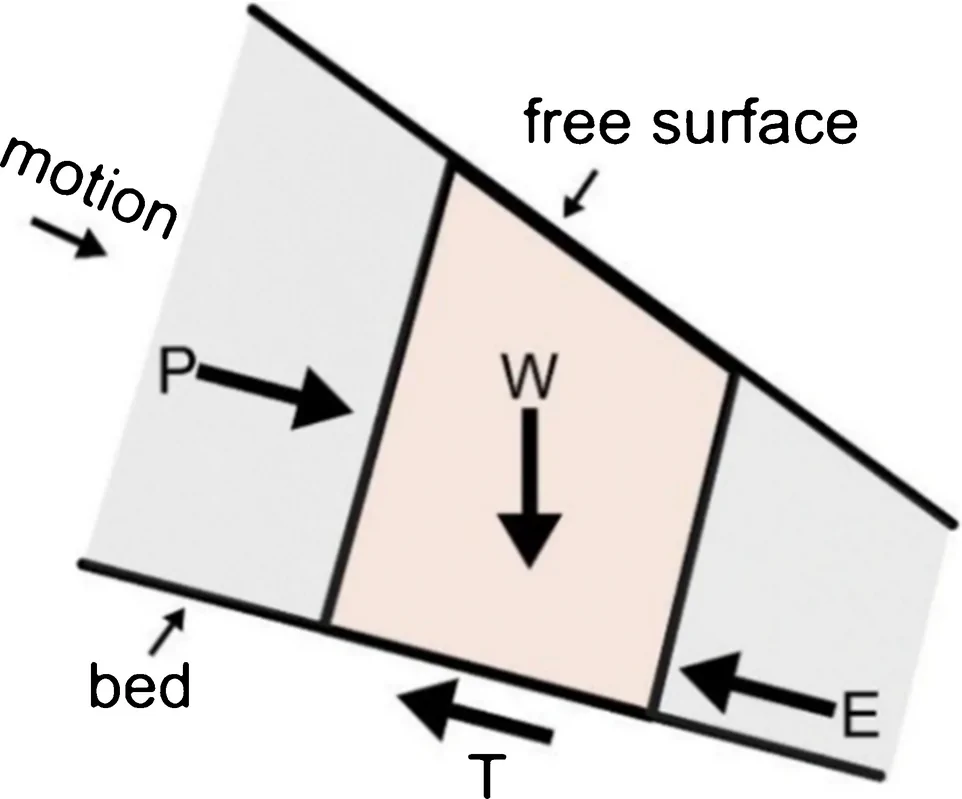
Conceptual illustration of the forces resolved by ORIN-3D. The slice is oriented in the direction of motion. W is the weight, T is the basal resistance, P is the internal force due to free surface gradients, and E is the inertial resistance due to entrainment (Aaron et al. 2017)

This work uses the GPU-accelerated numerical model ORIN-3D, which is a depth-averaged Lagrangian model that simulates landslide motion over complex, three-dimensional terrain, and is based on the model Dan3D (Three-Dimensional Dynamic ANalysis) (McDougall and Hungr 2004; Aaron et al. 2017). ORIN-3D is an SH (Savage-Hutter) type model, as the pressure term depends on the internal stress state (Savage and Hutter 1989, 1991; Hungr and McDougall 2009). ORIN-3D uses a numerical method based on SPH (Smoothed Particle Hydrodynamics) to solve the following equations of motion (Hungr and McDougall 2009):

1 $$\rho h\frac{D{v}_{x}}{Dt}=\rho h{g}_{x}-{k}_{x}{\sigma}_{z}\frac{\partial h}{\partial x}+{\tau}_{zx}-\rho E{v}_{x}$$

2 $$\rho h\frac{D{v}_{y}}{Dt}=\rho h{g}_{y}-{k}_{y}{\sigma}_{z}\frac{\partial h}{\partial y}$$

where ρ is the density, h is the flow depth, ${v}_{x}$ and ${v}_{y}$ are the depth-averaged velocities in the local x and y directions, respectively, ${g}_{x}$ and ${g}_{y}$ are the x and y components of gravity, ${k}_{x}$ and ${k}_{y}$ are the x and y lateral stress ratios (ratio of lateral stress to bednormal stress), ${\sigma}_{z}$ is the bed-normal stress, ${\tau}_{zx}$ is the basal resistance stress, and E is the entrainment ratio that governs momentum transfer when accelerating stationary bed material to the velocity of the moving Lagrangian reference frame. The forces resolved by ORIN-3D are shown in Fig. 3.

ORIN-3D adopts simplified rheological relationships based on the “equivalent fluid concept” (Hungr 1995). The parameters used in these rheologies cannot be measured using routine geotechnical testing and are instead constrained through back analysis of actual landslide case histories. The ${\tau}_{zx}$ in Eq. (1), the basal resistance stress, can be expressed in terms of a variety of rheologies; this work will mainly focus on Frictional and Voellmy rheology, since most of the case histories used in this work have the path materials of bedrock or sediments (saturated/unsaturated). The Frictional rheology is given by Hungr (1995):

3 $${\tau}_{zx}=-{\sigma}_{z}\mathrm{tan}\left({\phi}_{b}\right)$$

where ${\sigma}_{z}$ is the bed-normal effective stress and ${\phi}_{b}$ is the calibrated bulk friction angle, which includes pore-pressure effects. The Voellmy rheology is similar to the Frictional rheology, with an additional velocity-dependent term (Voellmy 1955):

4 $${\tau}_{zx}=-\left({\sigma}_{z}f+\frac{\rho g{{v}_{x}}^{2}}{\xi }\right)$$

where f is the friction coefficient (equivalent to $\mathrm{tan}\left({\phi}_{b}\right)$) and ξ is the turbulence coefficient. Both f and ξ are calibrated parameters. For the simulation of rock avalanches in ORIN-3D, when using these two types of rheology simultaneously, the Frictional rheology is usually used in the source area and the Voellmy rheology is used along the path (e.g., Figure 1B, Aaron et al. 2022).

### Calibration framework

Propagating both calibration and mechanistic uncertainties through the forecasting approach requires a calibration framework capable of quantifying calibration errors. Field investigations of specific landslide case histories enable the observation of key features that characterize an event such as impact area, velocity, deposit depth, and deposit distribution. The K observed features of an event can be represented by the following vector (Aaron et al. 2019):

5 $${{\boldsymbol{y}}}^{F}={\left({y}^{F,1}, \dots ,{y}^{F,K} \right)}^{T}$$

In ORIN-3D, given the values of the model parameter vector b, the corresponding simulation can be performed. The simulation outputs the simulated values of K observed features for a given case history, which can be represented by the following vector:

6 $${{\boldsymbol{y}}}^{M}\left({\boldsymbol{b}}\right)={\left({y}^{M,1}\left({\boldsymbol{b}}\right), \dots ,{y}^{M,K} \left({\boldsymbol{b}}\right)\right)}^{T}$$

The goal of model calibration is to determine the input parameter vector ${\boldsymbol{b}}$ so that the differences/residuals ${\boldsymbol{r}}\left({\boldsymbol{b}}\right)$ between the observed features ${{\boldsymbol{y}}}^{F}$ and the simulated features ${{\boldsymbol{y}}}^{M}\left({\boldsymbol{b}}\right)$ of an event are minimized:

7 $${\boldsymbol{r}}\left({\boldsymbol{b}}\right)={\left({r}^{1}\left({\boldsymbol{b}}\right), \dots ,{r}^{K}\left({\boldsymbol{b}}\right)\right)}^{T}$$

In this work, the impact area, which is the area enclosed by trimline, is used as the feature for calibration. The observed/measured trimline delineates the outer boundary of the rock avalanche impact over the entire event, as determined from field investigations. In contrast, the simulated trimline corresponds to the outermost contour where the simulated rock mass depth reaches or exceeds 1 m during the simulation. Fitness number ${F}_{trimline}$, which is the sum of the comparison between grids of simulation and measurement (trimline), is used to evaluate the simulation results of the impact area. When the simulation result at a grid point is consistent with the measurement, a value of 0 is assigned; otherwise, a value of 1 is assigned. The smaller the fitness number is, the better the simulation result is (Aaron et al. 2019).

There are some uncertainties in both the measured and simulated values of the observed landslide features, mainly due to the errors caused by feature measurement uncertainty (Prochaska et al. 2008; Aaron et al. 2019; Åberg et al. 2024), input data uncertainty, and the fact that the numerical model makes many simplifying assumptions, which together form the calibration uncertainty, which are quantified through a posterior probability density function, as defined below. A natural assumption is that the residuals ${\boldsymbol{b}}$ follow a multivariate Gaussian (or normal) distribution with a mean of zero and a covariance matrix of Σ. Under this normality assumption, the following likelihood function can be defined, which has a maximum value when the residual is zero (Hsieh 2009):

8 $$L\left({\boldsymbol{b}}|{{\boldsymbol{y}}}^{F},{{\boldsymbol{y}}}^{M}\left({\boldsymbol{b}}\right)\right)=\frac{1}{\sqrt{{\left(2\pi \right)}^{K}\left|\Sigma \right|}}\mathrm{e}\mathrm{x}\mathrm{p}\left(-\frac{1}{2}{{\boldsymbol{r}}\left({\boldsymbol{b}}\right)}^{T}{\Sigma }^{-1}{\boldsymbol{r}}\left({\boldsymbol{b}}\right)\right)$$

The parameter vector ${\boldsymbol{b}}$ that minimizes the difference between the observed and simulated measurements maximizes this likelihood function. In this paper, Bayesian posterior analysis is used to determine the parameter set of the maximization of the likelihood function (Aaron et al. 2019).

The posterior density of the calibrated parameters can be expressed using Bayes’ Law (Gregory 2010):

9 $${\pi}_{post}^{c}\left({\boldsymbol{b}}|{\boldsymbol{r}}\right)=\frac{L\left({\boldsymbol{b}}|{{\boldsymbol{y}}}^{F},{{\boldsymbol{y}}}^{M}\left({\boldsymbol{b}}\right)\right){\pi}_{prior}\left({\boldsymbol{b}}\right)}{\int \left({{\boldsymbol{b}}}^{\boldsymbol{*}}|{{\boldsymbol{y}}}^{F},{{\boldsymbol{y}}}^{M}\left({{\boldsymbol{b}}}^{\boldsymbol{*}}\right)\right){\pi}_{prior}\left({{\boldsymbol{b}}}^{\boldsymbol{*}}\right)d{{\boldsymbol{b}}}^{\boldsymbol{*}}}$$

where ${\pi}_{prior}$ is the prior density of ${\boldsymbol{b}}$; it represents prior belief about the possible values of the parameter vector ${\boldsymbol{b}}$ based on previous successful back-analyses. ${\pi}_{post}^{c}$ represents the posterior density after considering the available data for the case history and quantifies uncertainties associated with calibration and model simplifications (calibration uncertainty). Based on prior experience, ${{\boldsymbol{b}}}^{\boldsymbol{*}}$ is assumed to take value in region B, and with normally distributed errors, the posterior density is:

10 $${\pi}_{post}^{c}\left({\boldsymbol{b}}|{\boldsymbol{r}}\right)=\frac{\mathrm{e}\mathrm{x}\mathrm{p}\left(-\frac{1}{2}{\sum}_{k=1}^{K}\frac{1}{{{\sigma}_{k}}^{2}}{\left({r}^{k}\left({\boldsymbol{b}}\right)\right)}^{2}\right)}{{\int}_{B}\mathrm{e}\mathrm{x}\mathrm{p}\left(-\frac{1}{2}{\sum}_{k=1}^{K}\frac{1}{{{\sigma}_{k}}^{2}}{\left({r}^{k}\left({{\boldsymbol{b}}}^{\boldsymbol{*}}\right)\right)}^{2}\right)d{{\boldsymbol{b}}}^{\boldsymbol{*}}}$$

where ${\int}_{B}{\pi}_{post}^{c}\left({\boldsymbol{b}}|{\boldsymbol{r}}\right)d{\boldsymbol{b}}=1$. The standard deviation of the impact area/trimline fitness metric can be estimated by the following equation (Aaron 2017):

11 $${\sigma}_{trimline}=\frac{0.25{N}_{ones}}{2}$$

where ${N}_{ones}$ is the number of ones in the trimline grid file.

This work calibrates the parameter(s) of three different model parameterizations: that is, Voellmy rheology, Frictional rheology, Voellmy (path zone) and Frictional rheology (source zone). The range of parameters in the three model parameterizations, based on previous successful back-analysis, is shown in Table 2.

**Table 2:** Calibrated parameters setting

Model parameterization	Parameter	Range	Steps	Number of simulations
**Frictional rheology**	${\phi}_{b}$ (°)	0.5 to 45.0	0.5	1980
**Voellmy rheology**	f	0.01 to 0.40	0.01	22,000
	ξ (m/s^2^)	100 to 2500	100	
**Voellmy and Frictional rheology**	${\phi}_{b}$ (°)	10.0 to 30.0	1.0	369,600
	f	0.01 to 0.40	0.01	
	ξ (m/s^2^)	100 to 2000	100	

### Forecast framework

To investigate the influence of mechanistic uncertainty on forecasts of rock avalanche motion, a forecasting method is required that can account for all event-specific factors that effect rock avalanche behavior. Furthermore, it should leverage information from the full event database to estimate both the probability of impact and the corresponding runout exceedance probability, which is the probability of an event going a specified distance or further. The outcome of an event of interest for a future rock avalanche is denoted by ${\boldsymbol{E}}$, and its forecast probability is (Aaron et al. 2022):

12 $${p}_{pred}\left({\boldsymbol{E}}|{{\boldsymbol{r}}}^{\left(1\right)}, \dots ,{{\boldsymbol{r}}}^{\left(N\right)}\right)=\int p\left({\boldsymbol{E}}|{\boldsymbol{b}}\right){\pi}_{post}^{c}\left({\boldsymbol{b}}|{{\boldsymbol{r}}}^{\left(1\right)}, \dots ,{{\boldsymbol{r}}}^{\left(N\right)}\right)d{\boldsymbol{b}}$$

where $p\left({\boldsymbol{E}}|{\boldsymbol{b}}\right)$ represents the probability of a future outcome for a given parameter vector and N is the number of case histories. Inside the simulated impact area, $p\left({\boldsymbol{E}}|{\boldsymbol{b}}\right)=1$, and outside the simulated impact area, $p\left({\boldsymbol{E}}|{\boldsymbol{b}}\right)=0$. ${\pi}_{post}^{c}\left({\boldsymbol{b}}|{{\boldsymbol{r}}}^{\left(1\right)}, \dots ,{{\boldsymbol{r}}}^{\left(N\right)}\right)$ is the parameter’s posterior density based on the evidence by the combination of available data of all events in the database.

Assuming that potential future events will behave similarly to a subset of the case histories, all case histories of the database can be partitioned into ${N}_{c}$ disjoint “classes,” referred as ${{\boldsymbol{c}}}^{\left(i\right)}$, for $i=1, \dots , {N}_{c}$. Each “class” represents a specific mechanism of movement that occurs along the path of a given case history, such as effects from confinement (Strom et al. 2019; Mitchell et al. 2020), interaction with path material (Hungr and Evans 2004; Aaron and McDougall 2019), or potentially fragmentation of the rock avalanche material (Davies et al. 1999). These mechanisms combine to form a posterior distribution of a specific case history. The probability of the case history to forecast falling into the class ${{\boldsymbol{c}}}^{\left(i\right)}$ as $p\left({{\boldsymbol{c}}}^{\left(i\right)}\right)$. Since these classes are disjointed, it follows that ${\sum}_{i=1}^{{N}_{c}}p\left({{\boldsymbol{c}}}^{\left(i\right)}\right)=1$ and the law of total probability can be used to break down the combined parameter’s posterior into:

13 $${\pi}_{post}^{cm}\left({\boldsymbol{b}}|{{\boldsymbol{r}}}^{\left(1\right)}, \dots ,{{\boldsymbol{r}}}^{\left(N\right)}\right)=\sum\nolimits_{i=1}^{{N}_{c}}{{\pi}_{post}^{c}}^{(i)}\left({\boldsymbol{b}}|{{\boldsymbol{r}}}^{\left(i\right)}\right)p\left({{\boldsymbol{c}}}^{\left(i\right)}\right)$$

where ${{\pi}_{post}^{c}}^{(i)}\left({\boldsymbol{b}}|{{\boldsymbol{r}}}^{\left(i\right)}\right)$ is the model parameter posterior distribution for class ${{\boldsymbol{c}}}^{\left(i\right)}$, and ${\pi}_{post}^{cm}$ quantifies not only calibration uncertainty, but also mechanistic uncertainty which associates with unknown fundamental mechanisms that govern rock avalanche motion. $p\left({{\boldsymbol{c}}}^{\left(i\right)}\right)$ can be set subjectively by determining the similarity between the case histories in the database and the case history to forecast, which is called the expert-based forecast method; it can be also set equal for each case history (Aaron et al. 2022). In addition, this work will use a quantitative method to set $p\left({{\boldsymbol{c}}}^{\left(i\right)}\right)$, which will be explained in the following section.

### Case weightings

As described above, $p\left({{\boldsymbol{c}}}^{\left(i\right)}\right)$ is used to account for site specific factors that may influence runout behavior. To constrain this vector, two scenarios are compared: equally weighted posteriors (EQL) and quantitative weighted posteriors (QUA). In the EQL scenario, the weight of every case history is set to the same. In the QUA scenario, features affecting rock avalanche motion are quantified, and weights are determined based on the similarity of the forecast case to case histories. Research shows that topographic confinements and substrate have a great impact on landslide travel distance (Aaron and McDougall 2019; Mitchell et al. 2020; Aaron et al. 2022); therefore, topographic confinement and substrate are used as reference features for QUA scenario and quantify these features with one-hot encoding, see Table 3 for the features and their one-hot codes.

**Table 3:** Features for QUA scenario and their one-hot codes

Feature	Descriptor	One-hot code
**Confinement**	Frontal	100
	Lateral	010
	Unconfined	001
**Substrate**	Saturated	1000
	Unsaturated	0100
	Bedrock	0010
	Glacier	0001

To get the weight of each case, first, the Euclidean distances between the forecast case and the case histories are calculated by the following equation (Moslem and Pilla 2023):

14 $${d}_{i,k}=\sqrt{\sum\nolimits_{k=1}^{m}{\left({{\boldsymbol{x}}}_{k}-{{\boldsymbol{y}}}_{i}\right)}^{2}}$$

where i refers to the case used to forecast, and m=N-1; ${{\boldsymbol{x}}}_{k}$ is the feature vector of the case histories and ${{\boldsymbol{y}}}_{i}$ is the feature vector of the case to forecast. For example, for a rock avalanche with a frontal confinement and a saturated substrate, its feature vector is (1,0,0,1,0,0,0) according to Table 3.

Then, similarity scores between the case to forecast and the cases used for forecasting are getting by:

15 $${S}_{i,k}=\mathrm{e}\mathrm{x}\mathrm{p}\left(-\frac{{{d}_{i,k}}^{2}}{2{{\sigma}_{S}}^{2}}\right)$$

where ${\sigma}_{S}$ is a parameter used to adjust the exponential change of the similarity scores with the Euclidean distances, and it is set to 0.8 based on preliminary testing.

Finally, $p\left({{\boldsymbol{c}}}^{\left(i\right)}\right)$ is obtained by the following equation:

16 $$p\left({{\boldsymbol{c}}}^{\left(i\right)}\right)=\frac{{S}_{i,k}}{{\sum}_{k=1}^{m}{S}_{i,k}}$$

### Forecast sets

With the calibration and forecast framework in place, different methodologies for parameterizing the numerical model and estimating the case similarity weights can now be tested. In the present work, four different rheological sets that have been proposed for forecasting rock avalanche motion under two different weighting scenarios are tested. These forecast sets are summarized in Table 4.

**Table 4:** Forecast sets used for pseudo forecasts

Forecast set (Abbr.)	Scenario description
Equal Frictional (EQL Fri)	• All cases given equal weight, posterior distributions from model parameterization with Frictional rheology used
Equal Voellmy (EQL Voe)	• All cases given equal weight, posterior distributions from model parameterization with Voellmy rheology used
Equal Frictional-Voellmy (EQL Fri-Voe)	• All cases given equal weight, 4D* posterior distributions from model parameterization with Frictional and Voellmy rheology used
Equal Frictional-Voellmy Separate (EQL Fri-Voe Sep)	• All cases given equal weight, friction angle of Frictional rheology is obtained by Eq. (17). The posterior distribution of Voellmy rheology is the distribution from the cross-section with the largest sum of the posterior distribution among the friction angle cross-sections in the 4D posterior distribution
Quantitative Frictional (QUA Fri)	• Cases weights using Eq. (16), posterior distributions from model parameterization with Frictional rheology used
Quantitative Voellmy (QUA Voe)	• Cases weights using Eq. (16), posterior distributions from model parameterization with Voellmy rheology used
Quantitative Frictional-Voellmy (QUA Fri-Voe)	• Cases weights using Eq. (16), 4D posterior distributions from model parameterization with Frictional and Voellmy rheology used
Quantitative Frictional-Voellmy Separate (QUA Fri-Voe Sep)	• Cases weights using Eq. (16), friction angle of Frictional rheology is obtained by Eq. (17). The posterior distribution of Voellmy rheology is the distribution from the cross-section with the largest sum of the posterior distribution among the friction angle cross-sections in the 4D posterior distribution

*4D refers to friction angle, friction coefficient, turbulence coefficient, and posterior density

In Table 4, two forecast sets entitled “Equal/Quantitative Frictional-Voellmy Separate” are defined following previously recommended practices, which prescribe using a volume-dependent friction angle in the source zone and Voellmy parameters from similar cases along the runout path (Aaron et al. 2022). To implement this, the friction angle of the forecast case is determined by the regression equation (Eq. (17)). This regression equation is obtained by fitting the friction angle and volume of cases from the database of Aaron and McDougall (2019). For the cases common to both our database and theirs (21 of the 25 cases in their database), the friction angle is replaced with the angle associated with the friction angle cross-section that yields the highest total sum in the 4D posterior distribution in our work. The information of friction coefficient and turbulence coefficient is derived from the friction angle cross-section with the highest posterior probability summation in the 4D posterior distribution for each case, and then, different weights are assigned to each case history under the two scenarios. Finally, the forecast posterior probability distribution of friction coefficient and turbulence coefficient is still obtained by Eq. (13).

17 $${y}_{{\phi}_{b}}=-4.1574{\mathrm{log}}_{10}{x}_{V}+48.788$$

where ${y}_{{\phi}_{b}}$ is the friction angle (°) of the source zone for the case to forecast, and ${x}_{V}$ is the volume of the rock avalanche (m^3^). The coefficient of determination *R*^2^ of Eq. (17) is 0.1728. The scatter plot of friction angle vs volume of the rock avalanche with the linear regression line is shown in Supplementary Information. The limited performance of the regression is mainly driven by high calibration uncertainty (e.g., Supplementary Fig. 6 and Supplementary Fig. 22), whereby similar posterior probabilities across a range of friction angles result in dispersed regression data and a reduced goodness of fit.

To investigate the influence of calibration and mechanistic uncertainty, as well as to evaluate the optimal parameterization strategy for forecasts, a leave-one-out cross-validation approach is adopted. In this procedure, pseudo forecasts are performed by systematically excluding individual cases, one at a time, in order to assess the performance of the probabilistic forecasting framework on the remaining data (Adin et al. 2024). Specifically, the posterior densities from the remaining case histories are used in Eq. (13) to compute the forecast posterior density for the excluded case history. Samples are then drawn from this forecast posterior density, and the exceedance probability is obtained following the procedure described below. Then, the observed impact area of the event to forecast is compared with the forecast (exceedance probability) by this framework. To implement this, parameters are sampled according to the forecast posterior density (10^5^ samples for Frictional rheology, Voellmy rheology; 3× 10^6^ samples for Voellmy and Frictional rheology. Here, a sample is defined as the simulated impact area obtained from a sampled set of rheological parameters.), and the results corresponding to the random sampling are combined to determine the exceedance probability.

### Exceedance probability evaluation

The observed trimline is used to evaluate the quality of each pseudo forecast. The pixel in trimline matrix is assigned a value of 1 inside the trimline and 0 outside, and this binary matrix is compared against the simulated exceedance probability, which ranges from 0 to 1. As greater emphasis is placed on correctly forecasting runout within the trimline, and the area inside the trimline is typically smaller than the area outside, an approach is required that assigns higher weight to successful forecasts within the trimline than to those outside. To achieve this, the Tversky loss function is employed, as it allows different weights to be specified for regions inside and outside the trimline, to address data imbalance. The Tversky loss function is given as (Salehi et al. 2017):

18 $${\mathcal{L}}_{Tversky}=1-\frac{{\sum}_{i=1}^{{N}_{p}}{y}_{i}{\widehat{p}}_{i}+1}{{\sum}_{i=1}^{{N}_{p}}{y}_{i}{\widehat{p}}_{i}+\alpha {\sum}_{i=1}^{{N}_{p}}{y}_{i}\left(1-{\widehat{p}}_{i}\right)+\left(1-\alpha \right){\sum}_{i=1}^{{N}_{p}}\left({1-y}_{i}\right){\widehat{p}}_{i}+1}$$

where ${y}_{i}\in \left\{\mathrm{0,1}\right\}$ is a binary label of trimline matrix, for pixel i with 1 being inside the trimline and 0 being outside the trimline, while ${\widehat{p}}_{i}\in \left[\mathrm{0,1}\right]$ refers to the forecast exceedance probability of pixel i. ${N}_{p}$ is the total number of pixels of the trimline matrix or exceedance probability matrix. $\alpha \in \left[\mathrm{0,1}\right]$ is the weight added to the false negatives ${y}_{i}\left(1-{\widehat{p}}_{i}\right)$, and $\left(1-\alpha \right)\in \left[\mathrm{0,1}\right]$ is the weight added to the false positives $\left({1-y}_{i}\right){\widehat{p}}_{i}$; ${y}_{i}{\widehat{p}}_{i}$ refers to true positives. The range of the Tversky loss function is [0,1], and the smaller the value, the closer the forecast is to the true value. A further benefit of using the Tversky loss function instead of accuracy metrics like F1 score, recall, accuracy or IoU (Intersection over Union) score (Nguyen and Thai 2023) is that the Tversky loss function does not need to set a threshold to convert the forecast result to 0 or 1 before evaluation. In our simulation domains, the region with ${y}_{i}=0$ is usually several times larger than the region with ${y}_{i}=1$; α is set to 0.9 following tests of multiple alternatives informed by a subjective evaluation of the spatial agreement between the exceedance probability (particularly regions exceeding 0.5) and the observed trimline.

Finally, the Tversky loss function is evaluated for all case histories in the database using each of the forecast set listed in Table 4. Based on the resulting loss values, the forecast approach yielding the best simulation performance can be identified, along with any common characteristics among high-performing cases, such as volume, terrain confinement, or path material, that contribute to improved forecast quality.

## Results

### Calibration results

**Fig. 4:**
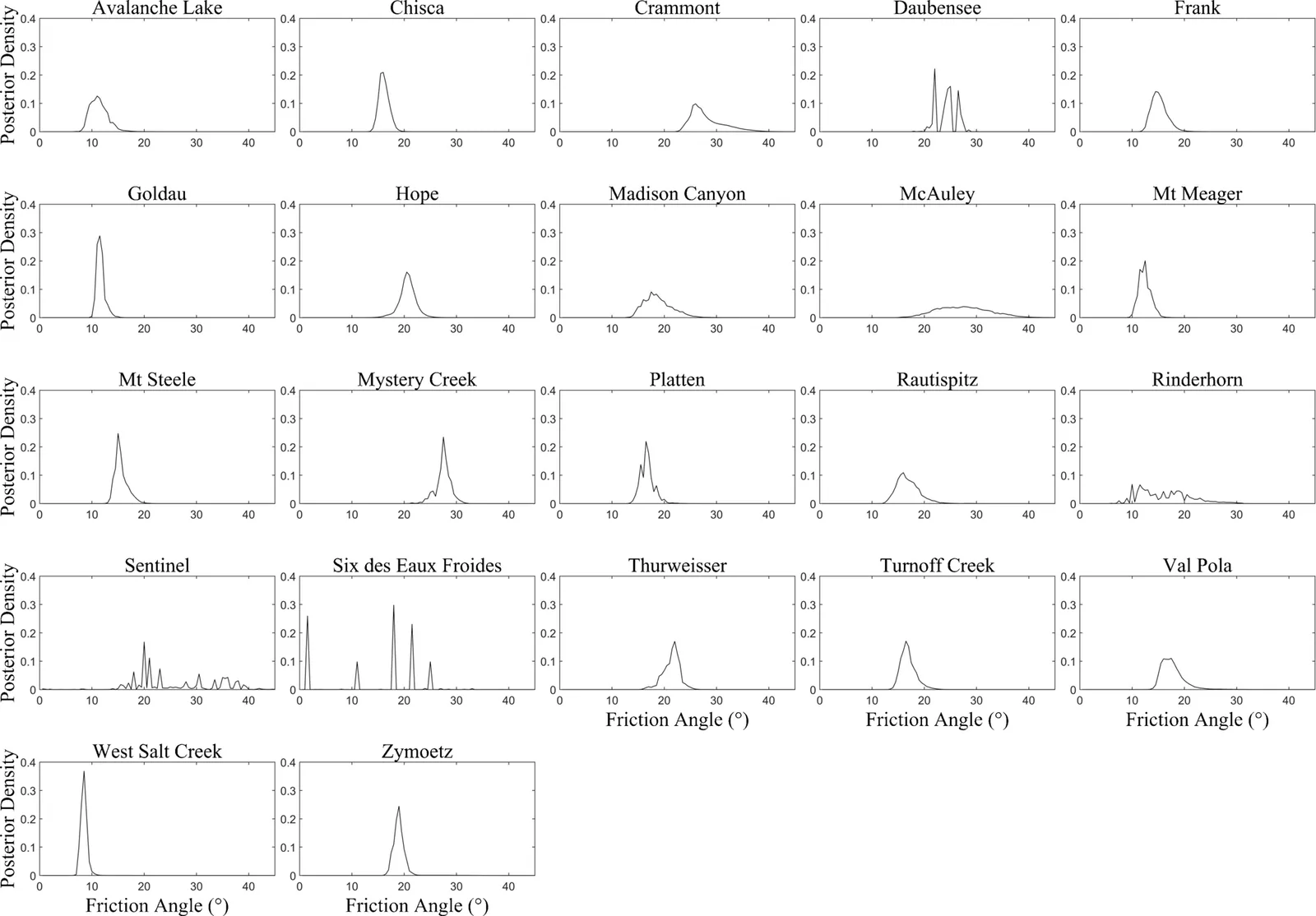
Posterior density of the case histories with Frictional rheology

Calibration uncertainty can be assessed by examining the posterior distributions that result from our calibration approach. Overall, we find that the posterior distributions vary significantly among the different case histories. Posterior distributions using the Frictional rheology are shown in Fig. 4. The majority of the posterior distributions are unimodal; some of them are high and narrow, such as Goldau, West Salt Creek, and some are wide and flat, such as Crammont, McAuley. A few cases have multi-modal posterior distributions, such as Daubensee, Sentinel, and Six des Eaux Froides, whose posterior distributions are irregular. In most cases, high posterior probability distribution is concentrated in the friction angle of 10 to 30°.

**Fig. 5:**
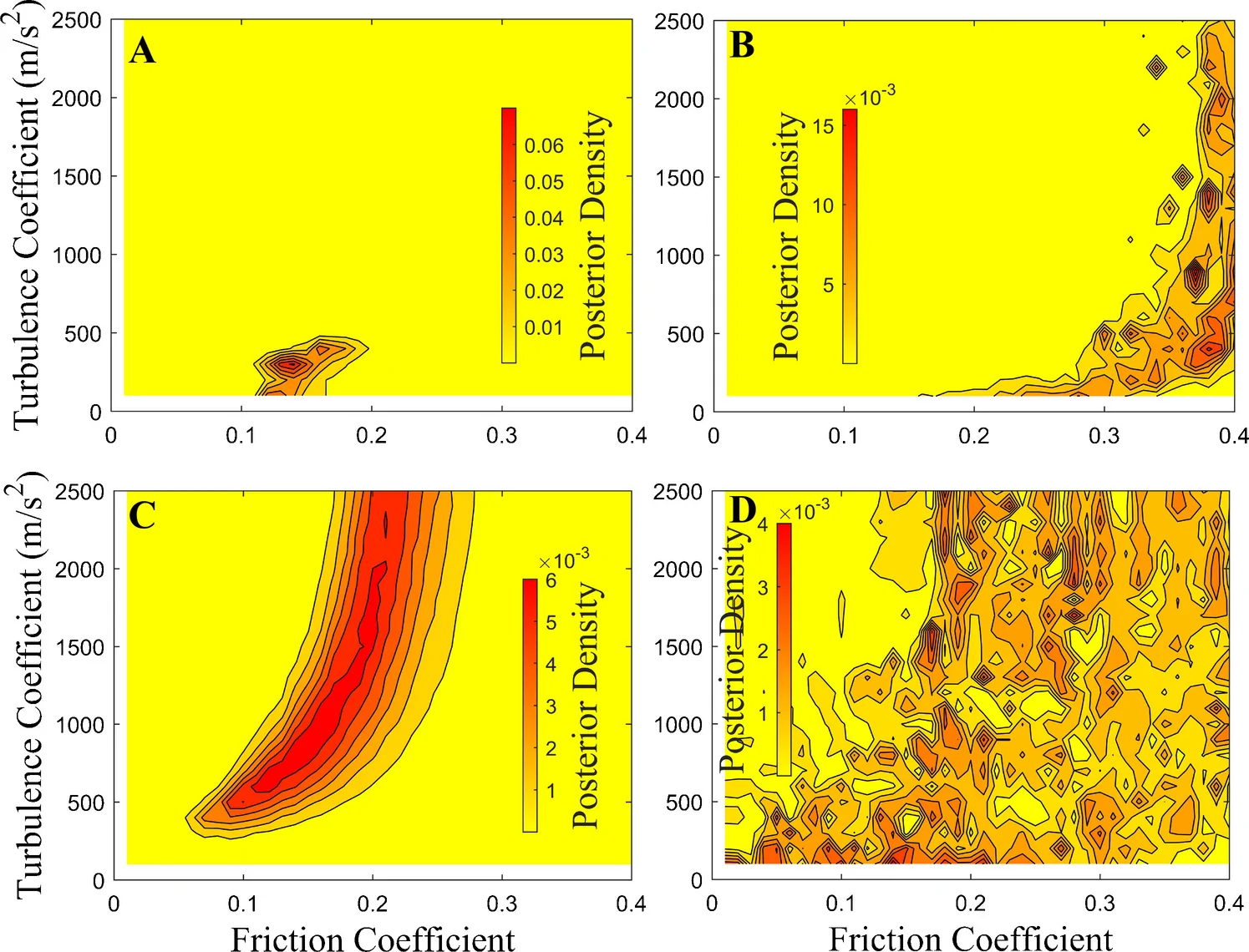
Posterior density of example case histories with Voellmy rheology. **A** Crammont. **B** Daubensee. **C** Frank. **D** Rinderhorn

When using the Voellmy rheology, we again find significant variation in the calibration results. The posterior distributions obtained using the Voellmy rheological parameterization can be classified into four types according to their shapes. Figure 5 shows one case of each of four types of the obtained distributions. The posterior distributions for the remaining cases are shown in Supplementary Information. Figure 5A shows the posterior distribution of Crammont, which is a concentrated type of distribution with one or a few peaks, cases with similar distribution shape including Goldau, Mt Meager and Zymoetz. Figure 5B shows the posterior distribution of Daubensee, which has multiple peaks in a banded area. Figure 5C shows posterior distribution of Frank, which is similar to the one shown in Fig. 5B, however with an absence of irregular peaks. The last distribution type is the irregular distribution shown in Fig. 5D of Rinderhorn. The results obtained using the Voellmy rheology thus indicate that parameter non-uniqueness is a common feature of this rheology and should be accounted for when forecasting.

**Fig. 6:**
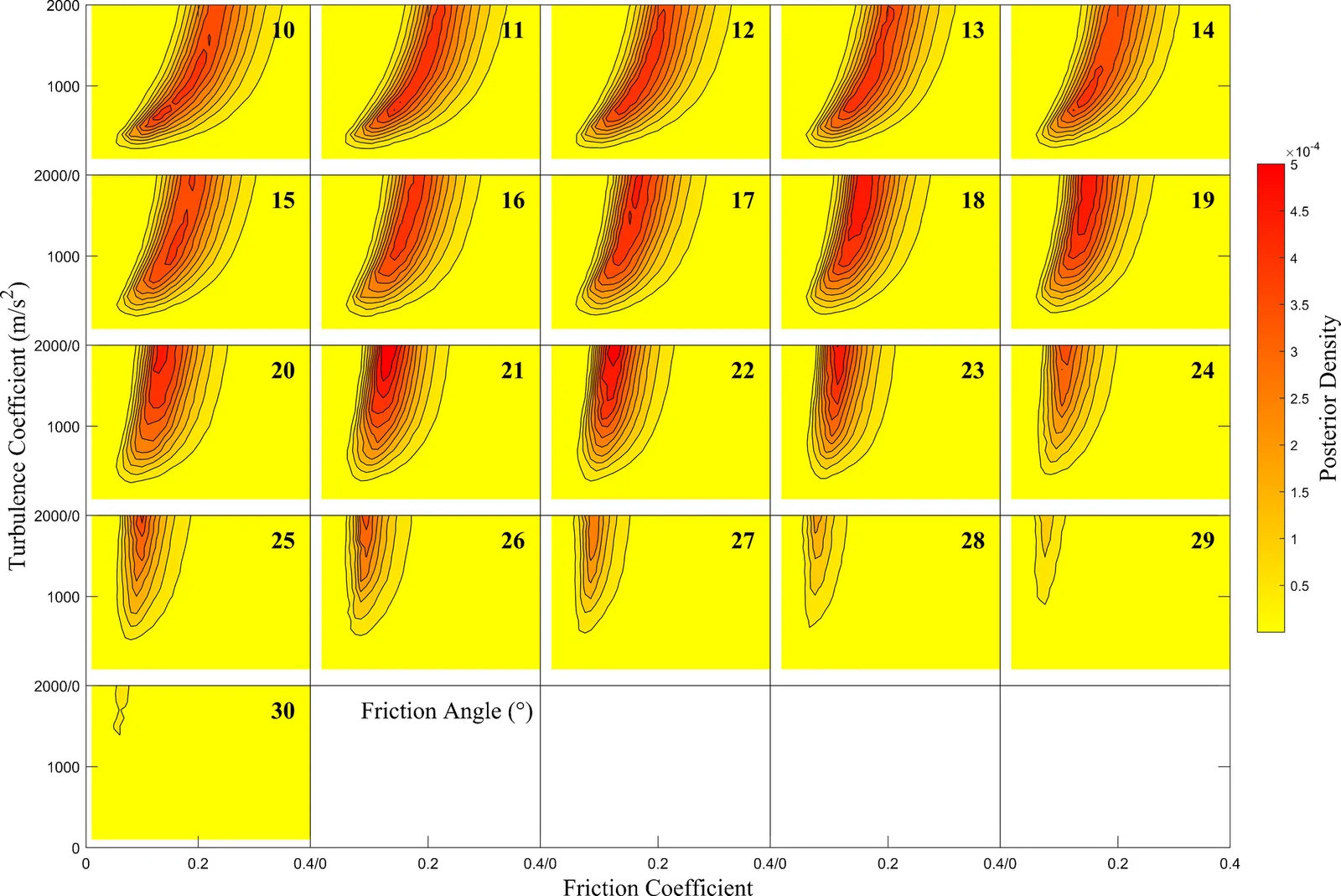
Posterior density of case Frank with Frictional and Voellmy rheology parameterization

The multi-rheology calibration results are similar to those obtained using only the Voellmy rheology. Figure 6 shows the posterior distribution of Frank, which is a typical posterior distribution type that is representative of many cases in the dataset. Figure 6 shows many friction angle cross-sections that have relatively high posterior probability distribution, and a similar appearance to Fig. 5C. Moreover, the distributions of each friction angle cross-section are very similar, indicating that the friction angle corresponding to these cross-sections has little influence on the simulation. A few other cases have a concentrated type of distribution, where the range of high posterior probabilities is concentrated in a tight space, such as Mystery Creek, and a few cases with an irregular type of distribution, such as the one of Daubensee (the Supplementary Information contains the posterior distributions for all cases). Taken together, these results indicate that calibration uncertainty is variable between the case histories and should be accounted for in forecasts.

### Example forecast of the Frank Slide

**Fig. 7:**
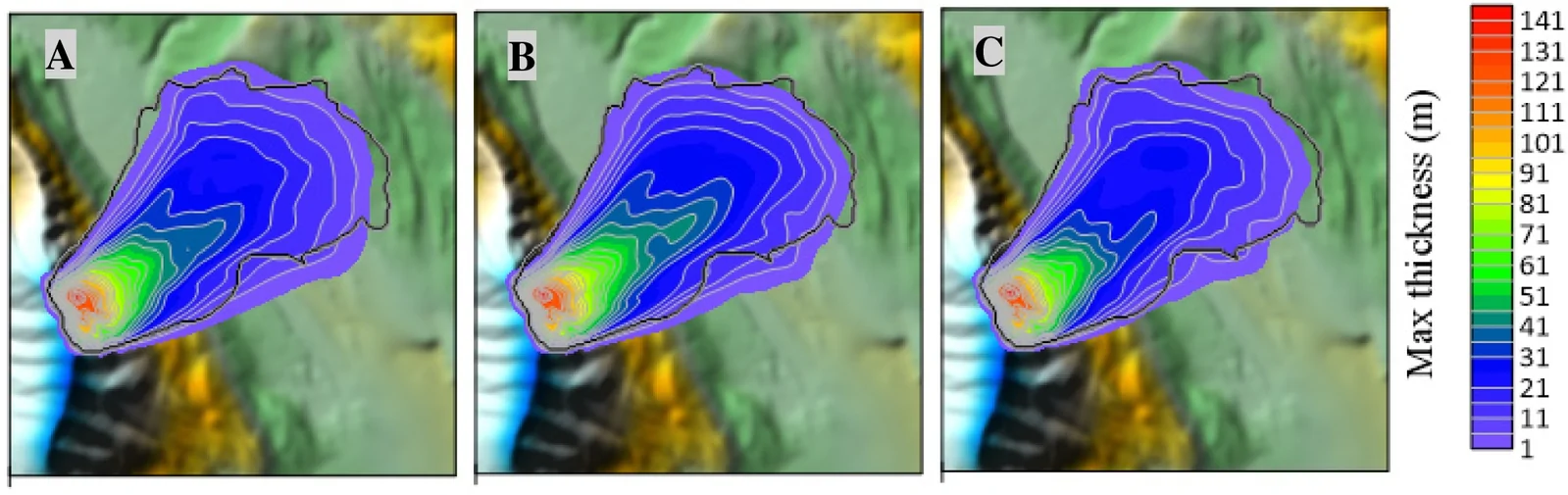
Best simulation of case Frank under **A** Frictional rheological parameterization, **B** Voellmy rheological parameterization, and **C** Frictional-Voellmy rheological parameterization. The black line is the measured trimline

In this section, we use the case of the Frank Slide as an illustrative example to demonstrate the complete workflow, from ORIN-3D simulations under different rheological parameterizations, to the posterior distributions inferred from these simulations, and finally to the forecast results under different forecasting sets, quantified as exceedance probabilities. Frank Slide is a tragic rock avalanche that occurred in Alberta, Canada, in 1903, killing about 70 people (Cruden and Krahn 1978). Figure 7 shows the best simulation using the max thickness (maximum “flow” thickness experienced during the simulation of the rock avalanche at each location) of case Frank under three parameterizations, which is evaluated by the Fitness number of trimline ${F}_{trimline}$. The best simulations produced by all three parameterizations are in strong concordance with the observed trimline.

**Fig. 8:**
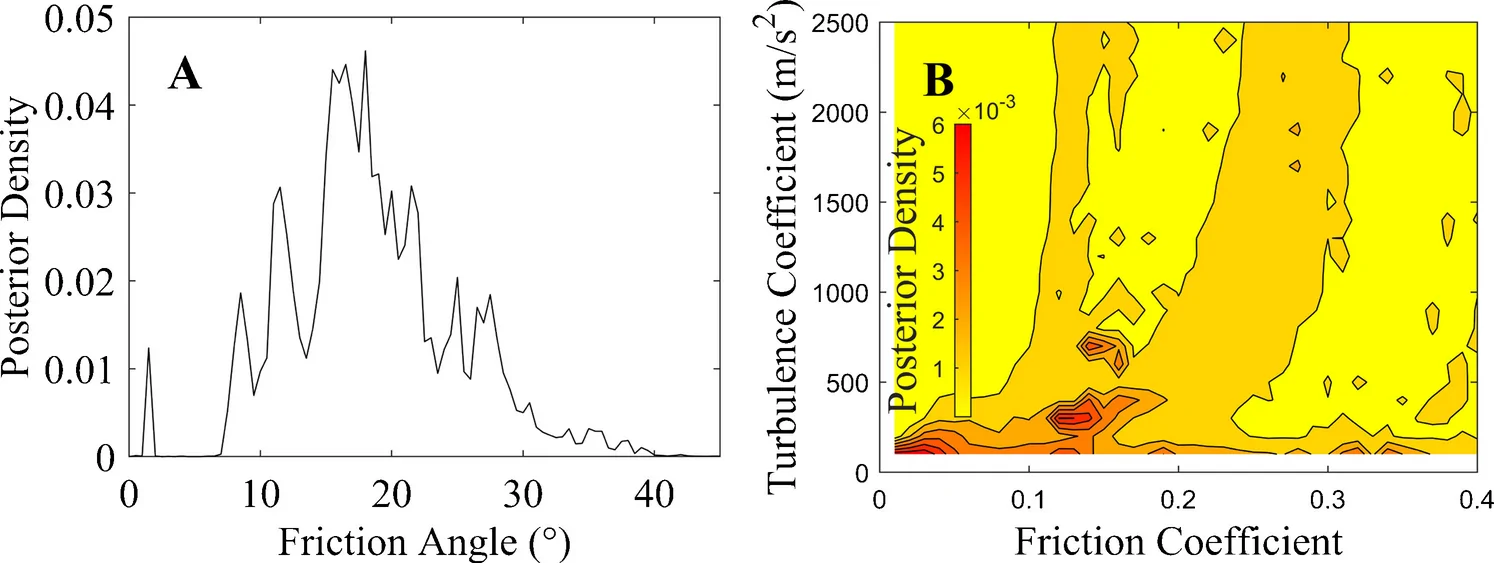
Forecast posterior density of case Frank under **A** Frictional rheological parameterization and **B** Voellmy rheological parameterization in EQL scenario

The first step of our forecasting approach involves obtaining a posterior distribution of the input rheological parameters to use for forecasting. Figure 8 shows the forecast posterior distributions of case Frank using the Frictional rheology to parameterize basal resistance, as well as the results obtained for the Voellmy rheological parameterization in EQL scenario (results obtained for the multi-rheology parameterization in EQL scenario are shown in Supplementary Information). It can be seen that those distributions are irregular, resulting from the variation in the distribution shape of each case history.

**Fig. 9:**
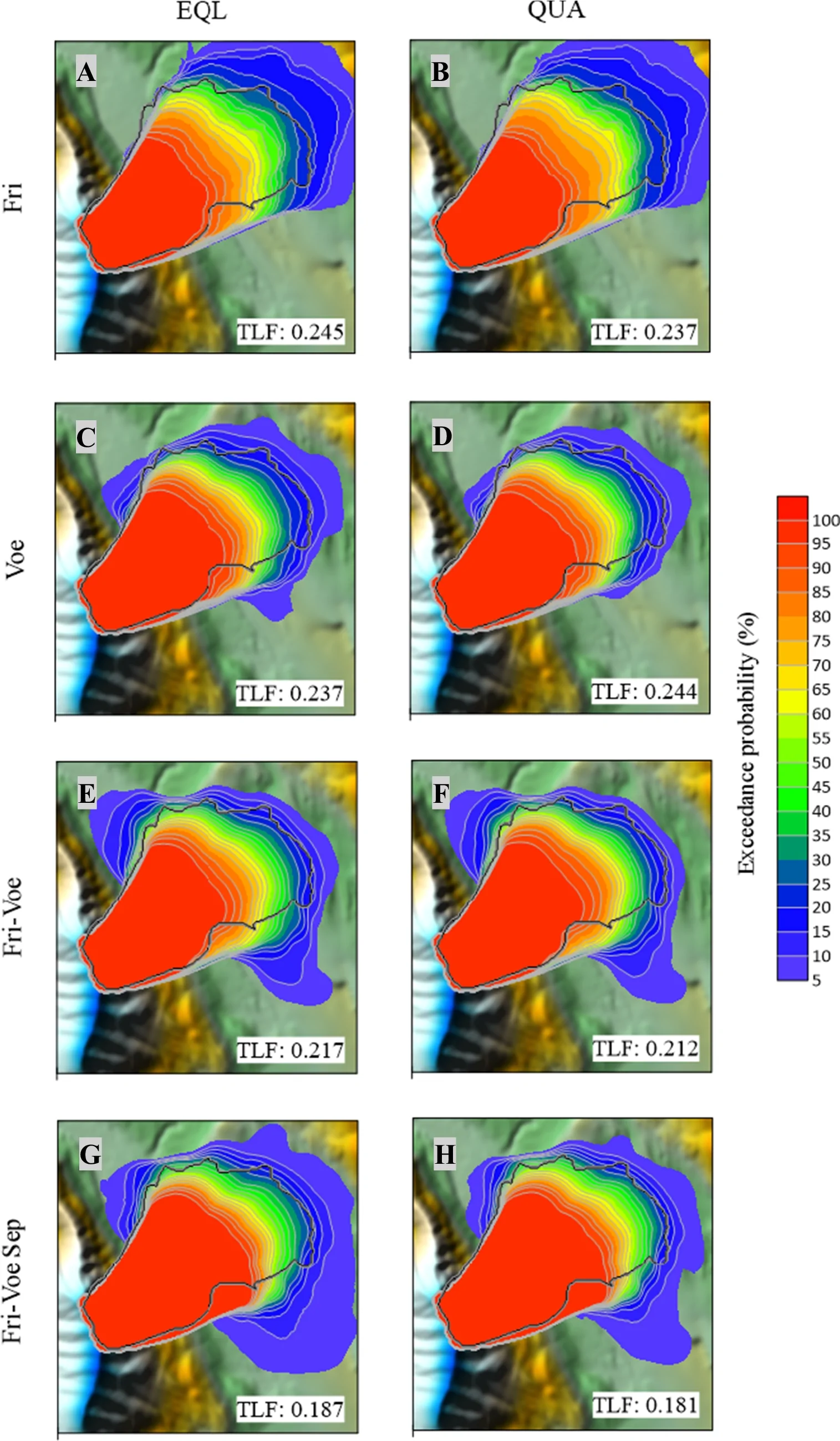
Exceedance probability of case Frank with eight forecast sets and their TLF values; the black line is the measured trimline

In the next step of the forecast, we obtain exceedance probability estimates for the potential impact area of the case history and use these to evaluate the TLF. The exceedance probabilities, as well as the TLF values of the eight forecast sets obtained for the Frank Slide, are shown in Fig. 9. For the EQL scenario, the Frictional rheology performs the worst, with a TLF value of 0.245, and the Frictional-Voellmy separate parameterization performs the best, with a TLF value of 0.187. For the QUA scenario, TLF has a slight but systematic improvement compared to EQL under the same parameterization sets. In the following section, we show how these example forecasts generalize to the entire database.

### Evaluation of all forecasts in the 22-event database

The goal of the present work is to understand the uncertainties inherent in forecasting rock avalanches of different types, using different model parameterizations. The results of applying our eight different forecasting parameterizations to the full rock avalanche dataset are shown in Table 5. The TLF values for each case history are arranged in ascending order, with the smallest being green, indicating the best forecast, and the largest being red, indicating the worst forecast. For these case histories, the worst forecast sets are generally the Frictional sets in EQL scenario; the best forecast sets are the Frictional-Voellmy separate sets in QUA scenario. For the vast majority of the case histories, under the same forecast set, the forecast results of QUA scenario are improved compared with the ones of EQL scenario.

**Table 5:**
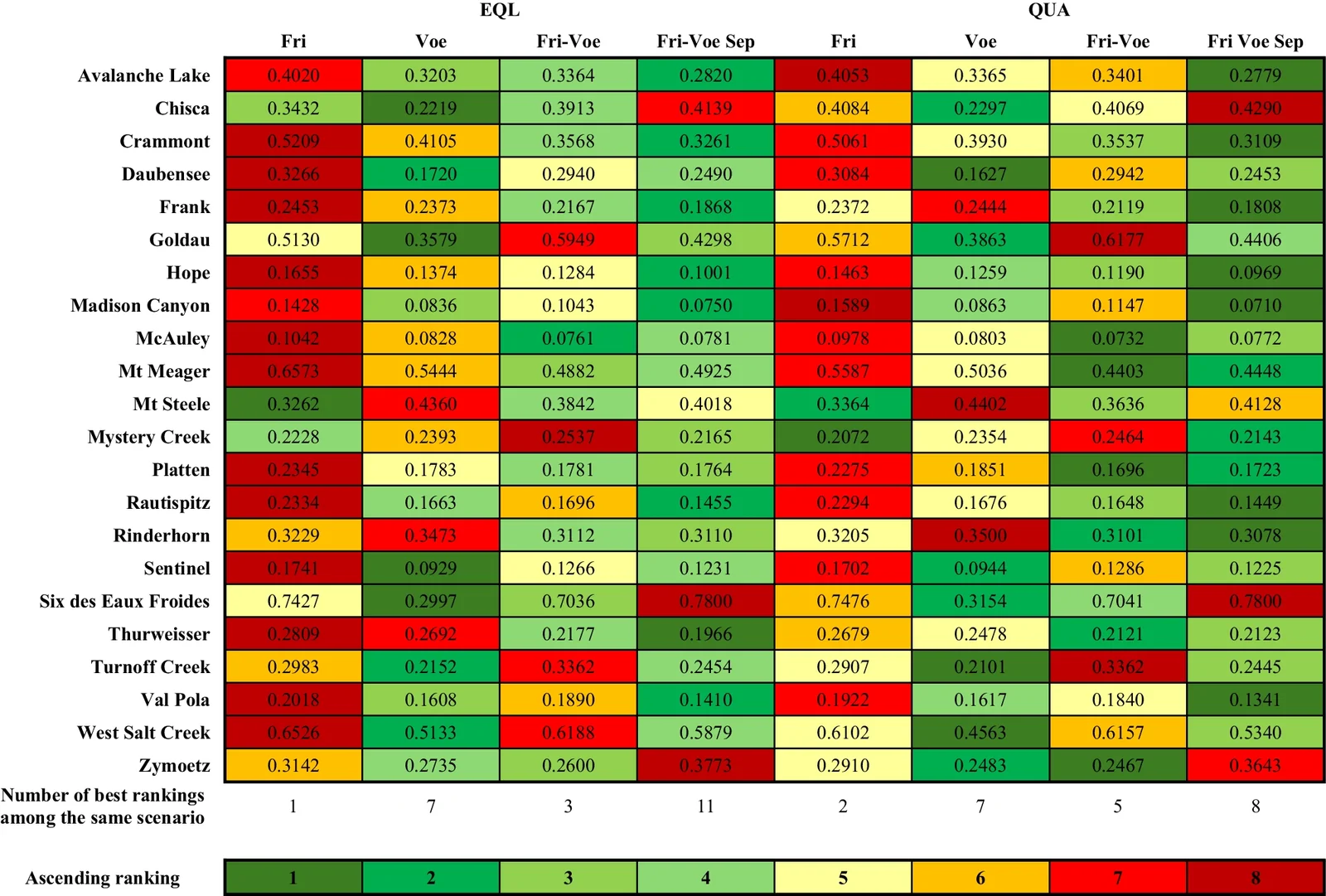
TLF value of the case histories under different forecast sets

We further explore this apparent forecasting improvement using the QUA scenario as opposed to the EQL scenario by quantifying the relative changes in TLF across different forecast sets. Table 6 shows that across different weighting scenarios, the TLF values in the QUA scenario decrease by only 1% to 2% compared with those in the EQL scenario, and a reduction is observed in only about half of the cases. The improvement using QUA is thus relatively small.

We also explored the influence of model parameterization on forecast performance. As shown in Table 6, the Voellmy rheology provides the greatest improvement, reducing the average TLF by about 20%, followed by the Frictional-Voellmy separate parameterization (17%) and the Frictional-Voellmy parameterization (10%). However, no uniform trend is observed across all case histories. While the Voellmy rheology provides the greatest overall improvement, individual cases respond differently to parameterization changes. For example, cases Daubensee and Mt Steele show contrasting behaviors, suggesting that the optimal parameterization may vary from case to case.

**Table 6:**
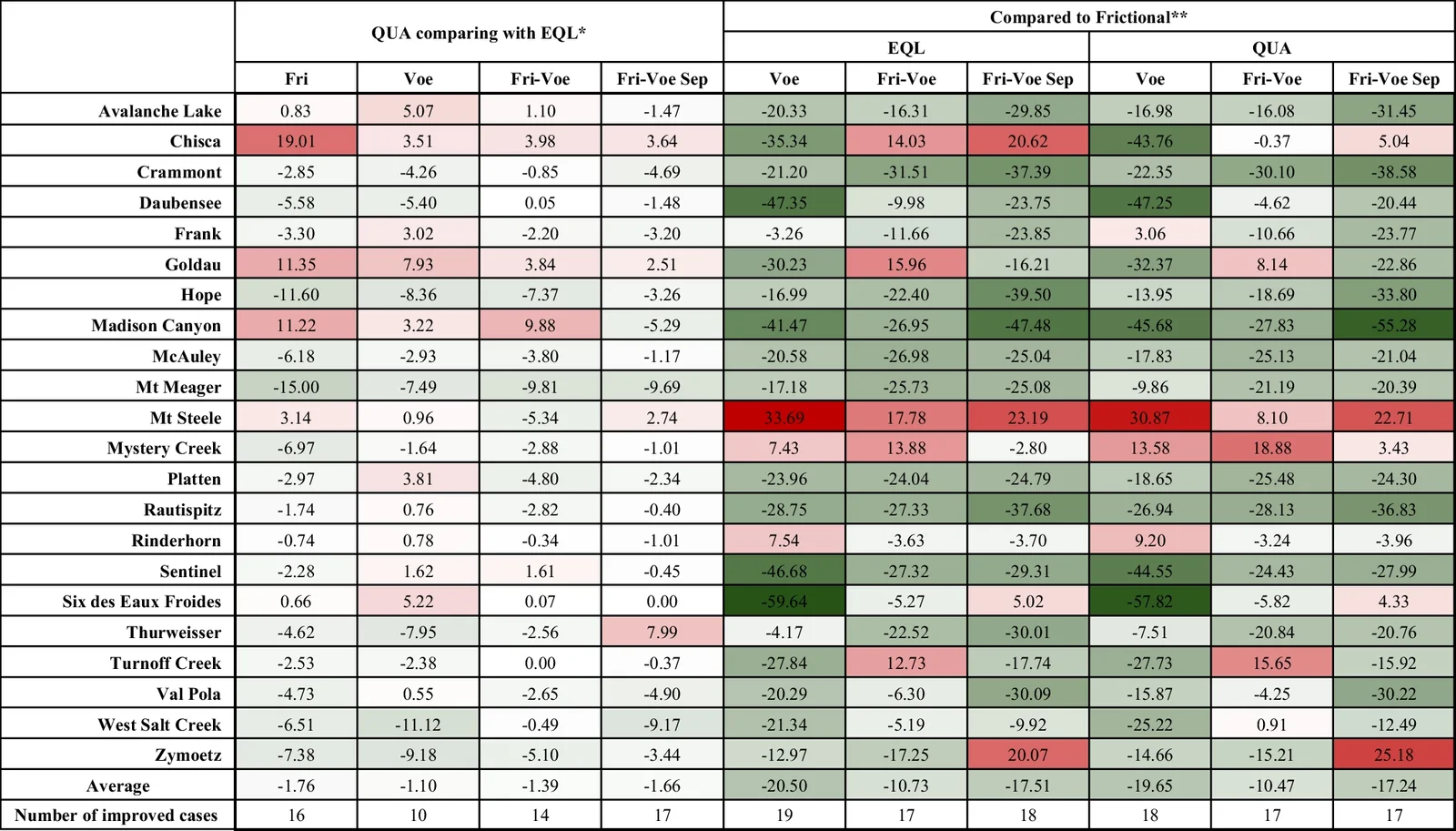
Percentage change in TLF value of different forecast scenarios for each case history

* ‘QUA compared with EQL’ refers to the percentage change in the TLF value of each case history in QUA scenario compared with the TLF value in EQL scenario under the same rheological setting, such as in Frictional rheological setting: 100×(TLF_QUA-Fri_- TLF_EQL-Fri_)/TLF_EQL-Fri_ ** ‘Compared to Frictional’ means the percentage change of the TLF value of Voellmy, Frictional-Voellmy and Frictional-Voellmy separate rheological setting compared to the TLF value of Frictional rheological setting in the same forecast scenario, such as in EQL scenario, the percentage change of the TLF value of Voellmy rheological setting compared to the TLF value of Frictional rheological setting: 100×(TLF_EQL-Voe_- TLF_EQL-Fri_)/TLF_EQL-Fri_

## Discussion

In the present work, we have leveraged GPU computing to perform a systematic evaluation of approaches for forecasting rock avalanche motion. Overall, we find that the highest forecasting accuracy is achieved when different rheologies are used in the source zone and along the path. Specifically, our leave-one-out cross validation approach applied to 22 case histories indicates that using a volume-dependent specification of the Frictional rheology in the source zone and Voellmy rheology along the path led to the best forecasting accuracy. This further supports research of Aaron and McDougall (2019), who found that source zone resistance is governed by a volume-dependent friction angle, but that resistance along the path is best captured with the Voellmy rheology. This was linked to the different materials overrun by the rock avalanche debris, and our results show that this mechanistic insight can be leveraged to increase forecasting accuracy, albeit with only a modest performance improvement (Table 6).

The use of GPU computing enables probabilistic assessment of a large ensemble of simulations. Obtaining this finding required around 400,000 individual simulations, which were used to obtain results for three different rheological parameterizations and 8 forecast sets. This was only possible using the recently developed numerical model ORIN-3D, which is optimized to run on a GPU. In fact, using a conventional model and single Intel i7 CPU, the simulation times would be on the order of 30 years. The approximately two orders of magnitude speedup obtained with ORIN-3D (Aaron 2023) has enabled a much larger parameter space to be explored, when compared with other methods that are used to overcome the high computational cost (Zeng et al. (2021), Sun et al. (2021a), Zhao and Kowalski (2022)).

Furthermore, the usage of GPU-based model also avoids the introduction of additional sources of uncertainty by solving the full equations of motion rather than using surrogate-based run-reducing strategies. For example, the Markov chain Monte Carlo simulation model of Zeng et al. (2021) has lower accuracy in the case of large terrain undulations due to non-linearities introduced by these topographic features, and the Gaussian process emulator of Yildiz et al. (2023) cannot handle uncertainty quantification for non-Gaussian distributions, such as the irregular distributions obtained in some of our case histories. We therefore expect that the use of a GPU-accelerated runout model enables more accurate forecasting and uncertainty quantification compared to these other methods to reduce runtime.

Our results have implications for understanding the influence of different sources of uncertainty on forecast outcomes. In particular, and as described in the methods, we distinguish calibration uncertainty and mechanistic uncertainty. The calibration results show that calibration uncertainty is variable among different parameterizations. The majority of posterior probability density functions using the Frictional rheology are unimodal (Fig. 4), or restricted to a well-defined range when using the Voellmy rheology (Fig. 5). These behaviors arise because calibration is based on a single trimline fitness number. While this information is sufficient to uniquely constrain the friction angle in the Frictional rheology, it is generally insufficient to independently constrain the two parameters of the Voellmy rheology, let alone the three parameters of Frictional and Voellmy rheology (Fig. 6). As a result, parameter non-uniqueness occurs, producing ridge-like posterior distributions. It thus appears that calibration uncertainty is minimized using the Frictional rheology; however the results (Table 6) show that the Voellmy rheology performs better for forecasting.

We found particularly high calibration uncertainty for a few cases, which resulted in irregular posterior distributions (Fig. 5D). These are mostly associated with pre-historic events, and the high calibration uncertainty is likely caused by uncertainties in the pre-event topography. It may be expected that these cases with high calibration uncertainty would have an outsized effect on forecast uncertainty. However, additional sensitivity analysis where case histories with irregular posterior densities (Daubensee, Rinderhorn, Sentinel, Six des Eaux Froides) are excluded (Supplementary Table 1) show that these cases have a negligible impact on forecast performance. This, combined with the discussion of the Frictional vs Voellmy rheology above, indicates that overall forecast uncertainty is not governed by calibration uncertainty.

Given the limited effect of calibration uncertainty, we hypothesize that mechanistic uncertainty dominates forecast uncertainty. We interpret this source of uncertainty to be the cause of the variation in the shape of the posterior distributions between cases (e.g., Figure 5, Supplementary Fig. 2 to 26). This is consistent with the findings of Aaron et al. (2022), who further suggested that mechanistic uncertainty can be addressed through appropriate weighting methods. However, in this work, the two weighting methods used (QUA and EQL) only produce slightly different results. The reason is that there are 21 case histories used for forecast, so the influence of the posterior distribution of each case history on the forecast posterior distribution is relatively small, regardless of EQL or the unequal-weighted QUA scenario. This shows that in the current forecast framework, the weight has little effect on the propagation of mechanistic uncertainty, as also illustrated by Aaron et al. (2022), unless mechanisms of all the case histories are well characterized. This finding differs from that of previous researchers, such as Zeng et al. (2021) and Zeng et al. (2022), as they used two consecutive landslides at the same location as past events and potential events, respectively, which minimize the impact of mechanistic uncertainties on forecasts.

Reducing mechanistic uncertainty requires that cases with different mechanisms can be assigned low weight when making forecasts. Achieving this depends on identifying the dominant mechanisms controlling the behavior of individual cases. We examined the TLF values of different case histories under the same forecast set to see if systematic trends between the best prediction set of each case history and the features (substrate material, terrain confinement, mobility) can be used to inform a more precise weighting strategy, therefore reducing mechanistic uncertainty (see also Supplementary Fig. 27 to 29). However, no convincing systematic trends emerged within our data set and analytical framework, despite only 1 of our 22 cases being classified as an outlier when compared to published mobility statistics (see “Data” section).

This is likely because the mechanistic uncertainties are caused by several factors that vary between events, and our dataset is not big enough to resolve this individual variation. One important factor is progressive disintegration of the rock avalanche material, which is not accounted for in our simulations nor incorporated into the QUA weighting approach adopted in this study. This process can limit lateral spreading, as has been previously found for the Mystery Creek and Goldau case histories (Aaron and Hungr 2016b). Furthermore, for some cases such as West Salt Creek, it is more suitable to use the Bingham rheology (Aaron et al. 2017), which was not considered here. Further research on rock avalanche mechanics is thus needed to reduce these uncertainties. In particular, we expect that performing back-analysis of a larger dataset of case histories, and interpreting the results in conjunction with established empirical correlations (Corominas 1996; Mitchell et al. 2020) will lead to improved methodologies for weighting the case histories.

Relatedly, in addition to applying appropriate weighting strategies, mechanistic uncertainty can be reduced by improving the numerical model itself. Simulating progressive fragmentation (e.g., using Dan3D Flex (Aaron and Hungr 2016b)), or alternatively developing a model capable of explicitly simulating the rock fragmentation process, is one promising future direction for model development. Furthermore, testing a wider range of rheologies (e.g., Figure 9, Table 6) may lead to improved forecast performance. These improvements, combined with the analysis of more case histories discussed above, are a promising path forward for improving forecasting performance, especially as more and bigger event inventories become available.

Our results have practical implications for forecasting rock avalanche motion, despite the uncertainties described above. They show that forecasts made with the Frictional rheology tend to underestimate the hazard in the distal part of the deposit within the trimline, overestimate in the distal part of the deposit outside the trimline (Fig. 9A and B), and that this can be steadily improved using the Voellmy rheology, and the combined Frictional-Voellmy parameterization (Table 5, Fig. 9C–H). This is of considerable importance for landslide risk analysis, as it facilitates the use of appropriate parameterizations based on both the relative location of the exposed elements with respect to the landslide source and their respective risk tolerance levels (e.g., a main railway corridor versus a rural road).

Furthermore, our results show that separating source and path resistance produces more accurate forecasts, albeit with a modest performance increase. However, this comes at the expense of having to specify additional parameters related to where to switch the basal rheology from Frictional rheology to Voellmy rheology, as also discussed in Aaron et al. (2022). Finally, we find that regardless of the parameterization used there is still significant uncertainty in the resulting forecasts, and the 5% or 10% exceedance probability zones can extend significantly beyond the observed trimline. This reflects mechanistic uncertainty that is difficult to reduce at present and highlights the significant uncertainties that should be expected when performing hazard analysis of rock avalanches.

Although the case histories in our database represent a broad sampling of the diversity of rock avalanche emplacement characteristics, the dataset is still limited in size. Path material has a significant influence on rock avalanche runout; however, the number of case histories for certain types, such as bedrock and glacier, is very small, which may further increase mechanistic uncertainty in forecasts. In addition, the calibration uncertainty varies among case histories, and for some case histories, it is quite large. These higher calibration uncertainties propagate through our forecast method to the case histories being predicted, resulting in degraded predictive performance. Nevertheless, the aforementioned limitations can be mitigated by expanding the dataset and improving understanding of individual rock avalanches. At the same time, our method becomes inefficient when handling high-dimensional parameter calibration and forecast, which may necessitate the use of surrogate modeling. However, this introduces additional uncertainty, and future work could explore appropriate strategies to achieve a balance between efficiency and accuracy.

## Conclusions

A Bayesian posterior analysis was performed to calibrate the GPU-accelerated runout model ORIN-3D for three rheological parameterizations: (1) Frictional, (2) Voellmy, and (3) a combined Frictional-Voellmy approach. Since we analyzed a dataset of 22 rock avalanche case histories, the analysis required around 400,000 simulations, which would have required decades of simulation time with conventional CPU models. Probabilistic forecasts for each case history were conducted using calibration results across eight forecast sets, and evaluated with the Tversky loss function. The results show that the best performing forecast methodology uses the Frictional-Voellmy separate parameterization, which confirms that in the source area, the volume of the rock avalanche affects the basal resistance, and when the rock avalanche leaves the source area, the influence of velocity-dependent resistance should be taken into consideration. Overall, we find that mechanistic uncertainty dominates forecast uncertainty, and future work focusing on the mechanics governing rock avalanche motion is required to improve forecast results, such as considering rock fragmentation, entrainment, and other bulk basal rheologies.

## Supplementary Information

Below is the link to the electronic supplementary material.

ESM 1 (DOCX 87.1 MB)

## Data Availability

The data supporting the findings of this study are not publicly available.
