# Supplementary material for: How well can rock avalanche runout be forecasted? GPU-Accelerated validation of a method for forecasting rock avalanche motion using a numerical model
Source: Landslides. 2026 Aug 26;23(11):3247–63. doi: 10.1007/s10346-026-02831-9 (PMC13669184; doi:10.1007/s10346-026-02831-9)
Supplement: Supplementary file 1 [file 10346_2026_2831_MOESM1_ESM.docx]

**How Well Can Rock Avalanche Runout be Forecasted? GPU-Accelerated Validation of a Method for Forecasting Rock Avalanche Motion using a Numerical Model**

**Guotong Deng, Jordan Aaron**

Guotong Deng (Corresponding Author)

Geological Institute, Department of Earth and Planetary Sciences, ETH Zürich, Zürich, Switzerland

guotong.deng@eaps.ethz.ch

The scatter plot of friction angle vs volume of the rock avalanche with the linear regression line is shown in Supplementary Fig. 1. The posteriori distribution of Voellmy rheology for each case history is shown in Supplementary Fig. 2 to 4. The posteriori distribution of Frictional and Voellmy rheology for each case history is shown in Supplementary Fig. 5 to 25. The predicted posterior distributions of case Frank under Frictional and Voellmy rheological parameterization in EQL scenario is shown in Supplementary Fig. 26. Scatter plots of H/L vs volume with point labels indicating the sets of the best prediction results for each case (QUA), path materials, and terrain confinements are shown in Supplementary Fig. 27 to 29.

**Supplementary Fig. 1** Scatter plot of friction angle vs volume of the rock avalanche with the linear regression line (Red line). Labels: 1. Avalanche Lake, 2. Chisca, 3. Crammont, 4. Daubensee, 5. Frank, 6. Goldau, 7. Hope, 8. Madison Canyon, 9. McAuley, 10. Mt Meager, 11. Mt Steele, 12. Mystery Creek, 13. Platten, 14. Rautispitz, 15. Rinderhorn, 17. Six des Eaux Froides, 18. Thurweisser, 19. Turnoff Creek, 20. Val Pola, 21. West Salt Creek, 22. Zymoetz, 23. Bingham Canyon, 24. Chehalis, 25. Guinsaugon, 26. Nomash

B

A

**Supplementary Fig. 2** Posterior density of the historical cases with Voellmy rheology. A: Avalanche Lake. B: Chisca.

B

A

D

C

F

E

H

G

**Supplementary Fig. 3** Posterior density of the historical cases with Voellmy rheology. A: Goldau. B: Hope. C: Madison Canyon. D: McAuley. E: Mt Meager. F: Mt Steele. G: Mystery Creek. H: Platten.

B

A

D

C

F

E

H

G

**Supplementary Fig. 4** Posterior density of the historical cases with Voellmy rheology. A: Rautispitz. B: Sentinel. C: Six des Eaux Froides. D: Thurweisser. E: Turnoff Creek. F: Val Pola. G: West Salt Creek. H: Zymoetz.

**Supplementary Fig. 5** Posterior density of case Avalanche Lake with Frictional and Voellmy rheology

**Supplementary Fig. 6** Posterior density of case Chisca with Frictional and Voellmy rheology

**Supplementary Fig. 7** Posterior density of case Crammont with Frictional and Voellmy rheology

**Supplementary Fig. 8** Posterior density of case Daubensee with Frictional and Voellmy rheology

**Supplementary Fig. 9** Posterior density of case Goldau with Frictional and Voellmy rheology

**Supplementary Fig. 10** Posterior density of case Hope with Frictional and Voellmy rheology

**Supplementary Fig. 11** Posterior density of case Madison Canyon with Frictional and Voellmy rheology

**Supplementary Fig. 12** Posterior density of case McAuley with Frictional and Voellmy rheology

**Supplementary Fig. 13** Posterior density of case Mt Meager with Frictional and Voellmy rheology

**Supplementary Fig. 14** Posterior density of case Mt Steele with Frictional and Voellmy rheology

**Supplementary Fig. 15** Posterior density of case Mystery Creek with Frictional and Voellmy rheology

**Supplementary Fig. 16** Posterior density of case Platten with Frictional and Voellmy rheology

**Supplementary Fig. 17** Posterior density of case Rautispitz with Frictional and Voellmy rheology

**Supplementary Fig. 18** Posterior density of case Rinderhorn with Frictional and Voellmy rheology

**Supplementary Fig. 19** Posterior density of case Sentinel with Frictional and Voellmy rheology

**Supplementary Fig. 20** Posterior density of case Six des Eaux Froides with Frictional and Voellmy rheology

**Supplementary Fig. 21** Posterior density of case Thurweisser with Frictional and Voellmy rheology

**Supplementary Fig. 22** Posterior density of case Turnoff Creek with Frictional and Voellmy rheology

**Supplementary Fig. 23** Posterior density of case Val Pola with Frictional and Voellmy rheology

**Supplementary Fig. 24** Posterior density of case West Salt Creek with Frictional and Voellmy rheology

**Supplementary Fig. 25** Posterior density of case Zymoetz with Frictional and Voellmy rheology

**Supplementary Fig. 26** Predicted posterior density of case Frank under Frictional and Voellmy rheological parameterization in EQL scenario

**Supplementary Fig. 27** Scatter plot of H/L vs volume with point labels indicating the sets of the best prediction results for each case (QUA)

**Supplementary Fig. 28** Scatter plot of H/L vs volume with point labels indicating path materials

**Supplementary Fig. 29** Scatter plot of H/L vs volume with point labels indicating terrain confinements

We also performed additional sensitivity analysis where case histories with irregular posterior densities (Daubensee, Rinderhorn, Sentinel, Six des Eaux Froides) are excluded. Supplementary Table 1 presents the percentage change in mean TLF values across forecast sets before and after exclusion, with both means consistently computed from the filtered case histories that exclude case histories exhibiting irregular posterior densities. It can be seen that case histories with irregular posterior densities have a negligible impact on forecast performance.

Supplementary Table 1. Percentage change in mean TLF values across forecast sets after excluding case histories with irregular posterior densities

|  | **EQL** | | | | **QUA** | | | |
| --- | --- | --- | --- | --- | --- | --- | --- | --- |
|  | **Fri** | **Voe** | **Fri-Voe** | **Fri-Voe Sep** | **Fri** | **Voe** | **Fri-Voe** | **Fri Voe Sep** |
| average | -5.46 | -5.14 | -3.27 | 0.63 | -3.87 | -2.61 | -1.02 | 1.20 |
